# Severe Polymicrobial Pneumonia With Septicemia After Interruption of Immunoglobulin Replacement in X‐Linked Agammaglobulinemia: A Case Report

**DOI:** 10.1155/crii/7974374

**Published:** 2026-09-10

**Authors:** Hau Dinh Tran, Quang Hung Vo, Nghia Phu Nguyen

**Affiliations:** ^1^ Department of Respiratory Medicine, Nhan Dan Gia Dinh Hospital, Ho Chi Minh City, Vietnam; ^2^ College of Health Sciences, Nam Can Tho University, 168 Nguyen Van Cu Street An Binh Ward, Can Tho City, Vietnam, nctu.edu.vn

**Keywords:** case report, immunoglobulin replacement therapy, *Klebsiella pneumoniae*, polymicrobial pneumonia, septicemia, *Streptococcus pneumoniae*, X-linked agammaglobulinemia

## Abstract

**Background:**

X‐linked agammaglobulinemia (XLA) is an inherited primary immunodeficiency characterized by impaired B‐cell maturation, profound hypogammaglobulinemia, and susceptibility to recurrent bacterial infections. Lifelong immunoglobulin replacement therapy (IGRT) is essential to reduce infectious complications, but treatment interruptions may markedly increase infection risk.

**Case Presentation:**

An 18‐year‐old male with XLA discontinued regular intravenous immunoglobulin (IVIG) replacement for 12 months because of financial constraints. He presented with fever, productive cough, pleuritic chest pain, hypoxemia, leukocytosis, and elevated inflammatory markers. His serum IgG level was 98.10 mg/dL. Chest computed tomography showed consolidation of the right middle and lower lobes without cavitation or bronchiectasis. Blood cultures and molecular testing of respiratory specimens identified both *Klebsiella pneumoniae* and *Streptococcus pneumoniae*, supporting polymicrobial pneumonia with bloodstream infection. A pathogenic Bruton tyrosine kinase (*BTK*) c.1522G >A (p.Ala508Thr) variant was confirmed. Intravenous imipenem–cilastatin and levofloxacin were administered according to antimicrobial susceptibility results. After 5 days, fever and oxygen dependence persisted despite improvement in leukocytosis. Following consultation with the hematology department, IVIG was readministered at 400 mg/kg, followed within 48 h by defervescence, discontinuation of supplemental oxygen, and marked improvement in inflammatory markers. He was discharged on hospital day 14, resumed IVIG every 4 weeks, and had no significant residual pulmonary lesion on follow‐up computed tomography 1 month later.

**Conclusion:**

This case highlights the consequences of interrupted immunoglobulin replacement in XLA and the importance of combining pathogen‐directed antimicrobial therapy with restoration of humoral protection in severe infection. It also illustrates how financial barriers may compromise continuity of lifelong treatment.

## 1. Introduction

X‐linked agammaglobulinemia (XLA) is an inborn error of immunity characterized by markedly reduced or absent mature B cells, severe hypogammaglobulinemia, and recurrent bacterial infections [1]. It is caused by mutations in the gene encoding Bruton tyrosine kinase (*BTK*), which is located on the X chromosome at Xq21.3‐q22 [1]. Loss‐of‐function BTK variants impair B‐cell receptor signaling and arrest B‐cell maturation, resulting in markedly reduced peripheral B cells and absent or severely reduced immunoglobulin production. While XLA is the most common cause of congenital agammaglobulinemia, accounting for ~85% of cases, other genetic forms have also been identified [2].

Patients with XLA commonly present with recurrent infections of the upper and lower respiratory tracts, including otitis media, sinusitis, and pneumonia, typically beginning at 6–9 months of age as maternally derived antibodies wane [1, 3]. Severe or life‐threatening infections, including pneumonia, empyema, meningitis, sepsis, cellulitis, and septic arthritis, often prompt evaluation for an underlying immunodeficiency [4]. Although most affected males present in infancy or early childhood, diagnosis may be delayed, particularly in patients with atypical or milder phenotypes [5]. The European Society for Immunodeficiencies (ESID) criteria for agammaglobulinemia include recurrent infections with onset before 5 years of age, serum IgG <500 mg/dL, age‐adjusted IgA and IgM levels more than two standard deviations below normal, and circulating B cells below 2% [6].

Because of severe antibody deficiency, patients with XLA are particularly susceptible to recurrent and invasive infections, especially those caused by encapsulated bacteria such as *Streptococcus pneumoniae* and *Haemophilus influenzae*. In the USIDNET Registry, respiratory infections were the most commonly reported infections among patients with XLA, affecting 203 of 231 patients (88%) [7]. A prior USIDNET analysis of 226 patients with XLA and known BTK mutations reported commonly identified organisms, including influenza B, *H. influenzae*, *Staphylococcus aureus*, *S. pneumoniae*, *Pseudomonas aeruginosa*, and *Giardia* [8]. In the Italian IPINet cohort of 168 patients with XLA [3], respiratory, gastrointestinal, and skin infections were prominent clinical manifestations; among pneumonia episodes with available sputum culture data, *H. influenzae* and *S. pneumoniae* were the most frequently isolated pathogens.

Severe invasive infections, including bacteremia/sepsis, meningitis, osteomyelitis, and septic arthritis, have been documented in patients with XLA [2]. Although regular immunoglobulin replacement therapy (IGRT) has reduced the incidence of invasive infections, these complications still occur and continue to contribute to morbidity and infection‐related mortality [3, 7]. We report a 18‐year‐old male with XLA who developed septicemia secondary to polymicrobial pneumonia caused by *Klebsiella pneumoniae* and *S. pneumoniae* after interruption of IGRT. This case highlights the importance of timely pathogen identification, appropriate antimicrobial therapy, reinitiation of immunoglobulin replacement, and long‐term strategies to protect lung function.

## 2. Case Presentation

An 18‐year‐old male was admitted to the hospital with a 5 day history of productive cough, pleuritic chest pain, and low‐grade fever up to 38.5°C. He denied dyspnea. There was no report of hemoptysis, weight loss, night sweats, recent tuberculosis exposure, or recent sick contact. His family history was unremarkable. His vaccination history was notable for influenza vaccination in the previous year, with no booster dose being administered before admission. The patient had been receiving regular intravenous immunoglobulin (IVIG) replacement therapy since his diagnosis of XLA at age 8 years, with IVIG administered every 4 weeks. However, treatment was discontinued 1 year before this hospitalization because of financial constraints. Baseline IgG trough levels before the treatment interruption were not available. His medical history was notable for three confirmed episodes of recurrent meningitis caused by *S. pneumoniae*, with the most recent infection occurring 3 years before admission. Records regarding IVIG adherence, dosing intervals, and trough IgG levels at the time of this most recent meningitis episode were not available.

On admission, his vital signs showed a temperature of 38.0°C, a heart rate of 105 beats/min, a respiratory rate of 26 breaths/min, and a blood pressure of 100/60 mmHg. Despite tachypnea and hypoxemia, he remained alert and hemodynamically stable, with an SpO_2_ of 90% while receiving supplemental oxygen via a nasal cannula at 2 L/min. Lung auscultation revealed fine crackles over the lower lung zones without wheezing.

Initial laboratory testing showed leukocytosis, with a white blood cell count of 18.05 × 10^9^/L and neutrophil predominance (76.2%), together with a markedly elevated C‐reactive protein level of 92.86 mg/L and a procalcitonin level of 0.82 ng/mL. Quantitative immunoglobulin testing was not obtained immediately after admission. Subsequent testing demonstrated profound hypogammaglobulinemia: IgG 98.10 mg/dL, IgM 16.3 mg/dL, IgA 5 mg/dL, and IgE 2.51 mg/dL. Blood cultures, as well as real‐time polymerase chain reaction analysis of bronchoalveolar lavage fluid and sputum, were positive for both *K. pneumoniae* and *S. pneumoniae*. Antimicrobial susceptibility testing showed susceptibility to imipenem–cilastatin and levofloxacin and resistance to piperacillin–tazobactam, ceftriaxone, penicillin, and erythromycin. Bronchial washings were negative for *Mycobacterium tuberculosis*, cytomegalovirus (CMV), influenza and COVID‐19. Direct sequencing confirmed a pathogenic *BTK* variant, c.1522G >A (p.Ala508Thr).

**Figure 1:**
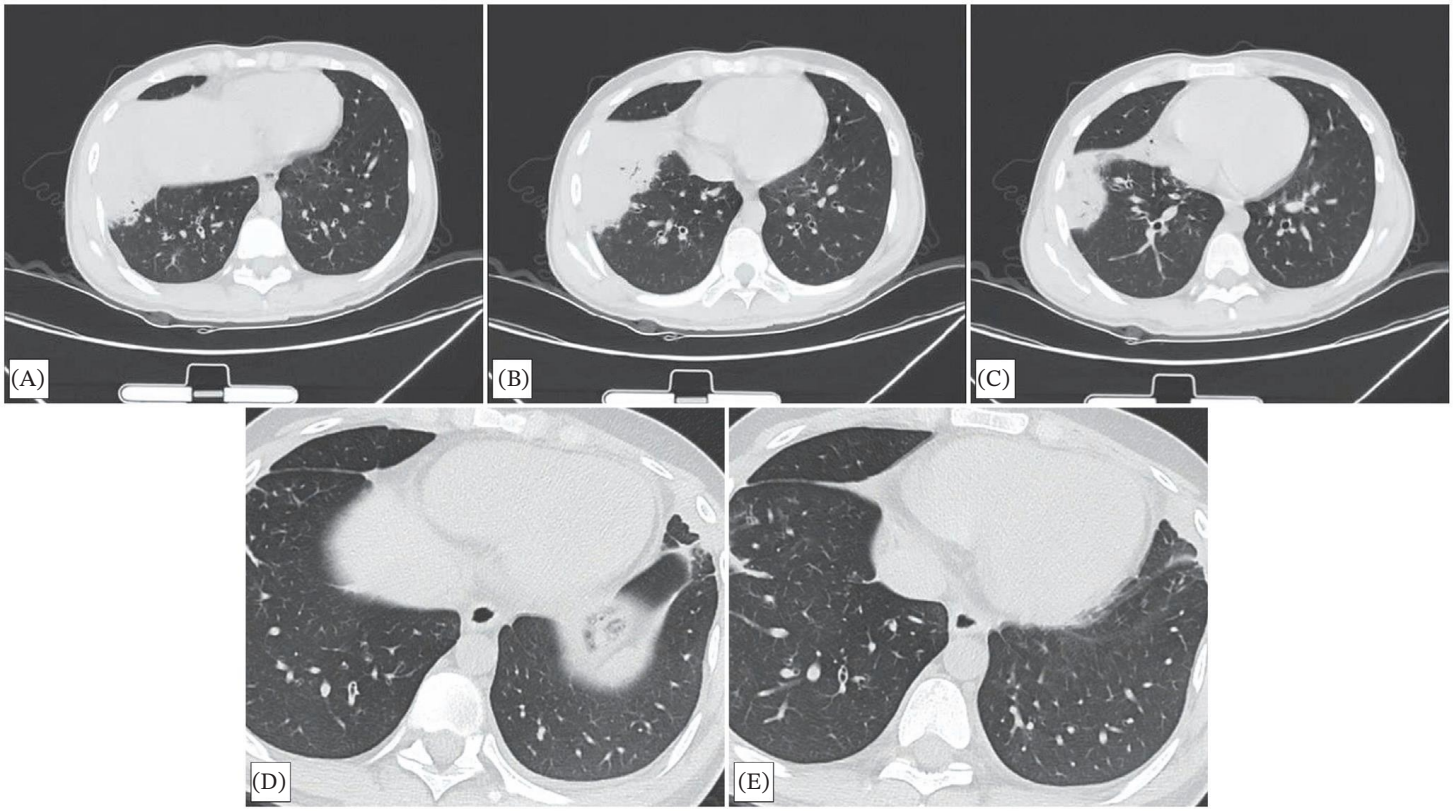
Chest CT images at presentation. Subfigures (A–C) show consolidation involving the right middle and lower lobes, including the posterior basal segment, consistent with pneumonia. No cavitation or bronchiectasis was observed. Subfigures (D, E) show follow‐up chest CT images obtained 1 month after discharge, demonstrating near‐complete resolution with no significant residual lesion in the right lower lung.

A transthoracic echocardiogram showed a left ventricular ejection fraction of 71%, with no regional wall motion abnormalities or signs of pulmonary hypertension. Chest radiography showed consolidation in the right lower lobe without pleural effusion. Subsequent chest computed tomography confirmed consolidation involving the right middle and lower lobes (Figure 1A–C).

The patient was initially treated with empirical intravenous imipenem–cilastatin 0.5 g every 4 h and levofloxacin 750 mg once daily. After 5 days of therapy, he showed no clear clinical improvement, with persistent fluctuating fever and ongoing oxygen requirement. Although the white blood cell count decreased from 18.05 × 10^9^ to 10.08 × 10^9^/L, inflammatory markers remained elevated, with a serum CRP level of 90 mg/L and a procalcitonin level of 0.8 ng/mL. As antimicrobial susceptibility testing confirmed susceptibility to the ongoing regimen, antibiotic therapy was continued.

Following consultation with the hematology department and in view of the persistent clinical manifestations and profoundly reduced immunoglobulin levels, IVIG was administered as a single dose of 400 mg/kg. Within 48 h, the patient improved markedly, with defervescence, resolution of oxygen requirement, and an SpO_2_ of 95% on room air. Inflammatory markers also decreased, with CRP falling by ~50% and procalcitonin decreasing to 0.18 ng/mL. He recovered uneventfully and was discharged on hospital day 14. No antibiotics were prescribed after discharge, and regular IVIG replacement every 4 weeks was resumed. Follow‐up chest imaging performed 1 month after discharge showed no significant residual lesion in the right lower lung (Figure 1D,E).

## 3. Discussion

This case highlights the importance of combining prompt antimicrobial therapy with reinitiation of IGRT in the management of severe infection in a patient with XLA. Patients with agammaglobulinemia are unable to generate effective pathogen‐specific antibody responses; therefore, regular IVIG or subcutaneous immunoglobulin replacement remains the cornerstone of long‐term management [9]. Although a trough IgG level of at least 500 mg/dL has historically been used as a minimum target [10], the adequacy of immunoglobulin replacement should be assessed using the trough IgG level together with the patient’s clinical course, and dose adjustment may be required for recurrent or excessive infections, growth, enteric loss, or increased immunoglobulin metabolism [2]. In this patient, a 12‐month interruption of IVIG was followed by a profoundly reduced IgG level of 98.10 mg/dL, likely leaving him with inadequate humoral protection against an invasive bacterial infection. In the USIDNET Registry, infection‐related mortality remained substantial among patients with XLA, with infection accounting for 12 of 17 deaths with a known cause (71%); chronic lung disease, often following repeated lower respiratory tract infections, was also associated with mortality [7]. The occurrence of severe polymicrobial pneumonia with septicemia after prolonged IVIG interruption is consistent with this increased susceptibility to serious infection.

The detection of *K. pneumoniae* and *S. pneumoniae* in blood cultures and respiratory specimens supported the diagnosis of polymicrobial pneumonia with bloodstream infection. *S. pneumoniae* is a typical encapsulated bacterial pathogen in patients with impaired humoral immunity, whereas *Klebsiella* species are recognized gram‐negative core respiratory pathogens in immunocompromised patients [11]. Antimicrobial susceptibility testing demonstrated susceptibility to imipenem–cilastatin and levofloxacin and resistance to piperacillin–tazobactam, ceftriaxone, penicillin, and erythromycin, supporting the continuation of the initial intravenous regimen. In hospitalized immunocompromised patients with pneumonia, a comprehensive microbiologic work‐up is recommended to identify the causative pathogens and facilitate pathogen‐directed therapy or de‐escalation, while the extent of testing should be individualized according to likely organisms, patient‐specific risk factors, and local diagnostic capabilities [11]. Although this patient remained alert and hemodynamically stable, immunocompromised patients with pneumonia may appear clinically stable at initial evaluation and subsequently deteriorate rapidly, supporting a low threshold for hospital admission [11]. In the setting of profound hypogammaglobulinemia and persistent clinical manifestations despite an active antimicrobial regimen, IVIG was reinitiated to restore antibody replacement. Clinical improvement followed IVIG administration; however, its specific contribution cannot be separated from the ongoing effect of antimicrobial therapy. In this case, limited experience with adolescent patients with known primary immunodeficiency in the admitting Respiratory Medicine service contributed to delayed quantitative immunoglobulin testing and IVIG reinitiation; treatment was initiated after consultation with the hematology department. This experience highlights the importance of early involvement of clinicians experienced in primary immunodeficiency when patients with XLA present with severe infection.

The financial circumstances surrounding the 12‐month interruption also illustrate a major practical barrier to sustained IGRT [2]. The patient reported a monthly income of ~10–12 million VND, whereas IVIG at 400 mg/kg every 4 weeks cost ~20–30 million VND per treatment and was not reimbursed under his insurance coverage. At this rate, 12 months of IVIG would cost ~240–360 million VND, compared with an out‐of‐pocket cost of ~2–3 million VND for this hospitalization after insurance coverage. Although this descriptive comparison does not constitute a formal economic analysis, it illustrates the substantial affordability barrier to preventive IGRT and the need for financial and logistical support for patients requiring lifelong replacement therapy.

Immunoglobulin replacement is the mainstay of treatment for XLA and is an important determinant of infection risk, although respiratory infections and chronic lung disease may still occur despite therapy [2]. The absence of bronchiectasis on initial CT and the resolution of pulmonary lesions at a 1‐month follow‐up were reassuring. Nevertheless, long‐term care should prioritize uninterrupted IGRT, assessment of IgG trough levels together with infection frequency, prompt evaluation of recurrent respiratory symptoms, and surveillance for bronchiectasis or other chronic pulmonary complications [2, 3].

Some limitations should be acknowledged. First, longitudinal data on IVIG adherence, dosing intervals, and IgG trough levels, including at the time of the pneumococcal meningitis episode 3 years earlier, were unavailable, precluding assessment of the adequacy of replacement therapy over time. Second, because antimicrobial therapy was continued when IVIG was administered, the rapid clinical improvement cannot be attributed specifically to IVIG reinitiation. Finally, follow‐up was limited to 1 month after discharge, and longer observation would be required to assess recurrent infections, sustained adherence to IGRT, and the development of chronic pulmonary complications.

## 4. Conclusion

Interruption of immunoglobulin replacement in XLA may result in profound hypogammaglobulinemia and severe invasive bacterial infection. This case underscores the importance of uninterrupted IGRT, prompt pathogen‐directed antimicrobial therapy, and long‐term follow‐up to prevent recurrent infection and chronic pulmonary complications. Early involvement of clinicians experienced in primary immunodeficiency and appropriate financial and logistical support may also be critical to avoiding treatment delays and maintaining lifelong IGRT.

## Author Contributions

Hau Dinh Tran and Quang Hung Vo collected and interpreted the clinical data and drafted the manuscript. Nghia Phu Nguyen critically revised the manuscript for important intellectual content and approved the final version for publication.

## Funding

No funding was received for this study.

## Disclosure

All authors have read and approved the final manuscript.

## Ethics Statement

The authors have nothing to report.

## Consent

Informed consent was obtained from the parents of the patient prior to being included in the study.

## Conflicts of Interest

The authors declare no conflicts of interest.

## Data Availability

Data sharing is not applicable to this article, as no datasets were generated or analyzed during the current study.
